# TIGIT Deficiency Protects Mice From DSS-Induced Colitis by Regulating IL-17A–Producing CD4^+^ Tissue-Resident Memory T Cells

**DOI:** 10.3389/fimmu.2022.931761

**Published:** 2022-07-01

**Authors:** Binfeng Chen, Baokui Ye, Mengyuan Li, Shuyi Wang, Jin Li, Yimei Lai, Niansheng Yang, Zunfu Ke, Hui Zhang

**Affiliations:** ^1^ Department of Rheumatology, The First Affiliated Hospital, Sun Yat-sen University, Guangzhou, China; ^2^ Department of Pathology, The First Affiliated Hospital, Sun Yat-sen University, Guangzhou, China; ^3^ Institue of Precision Medicine, The First Affiliated Hospital, Sun Yat-sen University, Guangzhou, China

**Keywords:** inflammatory bowel diseases, DSS-induced colitis, tissue-resident memory T cells, TIGIT, interleukin-17A

## Abstract

Tissue-resident memory T cells (T_RM_ cells) have been shown to play an instrumental role in providing local immune responses for pathogen clearance in barrier tissues. However, their contribution to inflammatory bowel diseases (IBDs) and the underlying regulation are less clear. Here, we identified a critical role of T-cell immunoreceptor with immunoglobulin and ITIM (TIGIT) in regulating CD4^+^ T_RM_ cells in an experimental model of intestinal inflammation. We found that CD4+ TRM cells were increased and correlated with disease activities in mice with dextran sulfate sodium (DSS)-induced colitis. Phenotypically, these CD4^+^ T_RM_ cells could be classified into CD69^+^CD103^−^ and CD69^+^CD103^+^ subsets. Functionally, these CD4^+^ T_RM_ cells were heterogeneous. CD69^+^CD103^−^ CD4^+^ T_RM_ cells were pro-inflammatory and produced interferon-γ (IFNγ) and interleukin-17A (IL-17A), which accounted for 68.7% and 62.9% of total IFNγ^+^ and IL-17A^+^ CD4^+^ T cells, respectively, whereas CD69^+^CD103^+^ CD4^+^ T_RM_ cells accounted for 73.7% Foxp3^+^ regulatory T cells. TIGIT expression was increased in CD4^+^ T cells in the gut of mice with DSS-induced colitis. TIGIT deficiency impaired IL-17A expression in CD69^+^CD103^−^ CD4^+^ T_RM_ cells specifically, resulting in ameliorated gut inflammation and tissue injury. Together, this study provides new insights into the regulation of gut inflammation that TIGIT deficiency protects mice from DSS-induced colitis, which might have a potential therapeutic value in the treatment of IBDs.

## Introduction

Inflammatory bowel diseases (IBDs) such as Crohn’s disease and ulcerative colitis are characterized by chronic inflammation of the gastrointestinal tract, followed by immune-induced tissue injury and disruption of epithelial barrier (1). The pathogenesis of IBDs is multifactorial, involving genetic susceptibility, environmental factors, microbiota–host immune interactions, and dysregulated mucosal immunity (2). Among them, T cells have been suggested as a key player in driving experimental murine colitis and human IBDs (3, 4).

Data have revealed that CD4^+^ (TH) cell–derived cytokines are important in the pathogenesis of IBDs (5). The roles of CD4^+^ TH cell subsets such as T_H_1, T_H_2, T_H_9, and T_H_17 cells have been demonstrated and featured as disease driver by producing pro-inflammatory cytokines (6–9). In contrast, regulatory T cells (T_reg_) exert anti-inflammatory function and provide a protective role in IBDs (10). Nevertheless, these studies focused on the functions and differentiation of circulating T cells in IBDs. Less is understood in regarding the function and regulation of T cells that reside in the gut and their association with the pathogenesis of IBDs.

Tissue-resident memory T cells (T_RM_ cells), an important memory T-cell subset, persist for a long term in epithelial barrier tissues and help maintaining local homeostasis (11, 12). T_RM_ cells are characterized by the expression of C-type lectin CD69, which counteracts sphingosine 1 phosphate receptor 1–mediated tissue egress and promotes tissue residency (13). T_RM_ cells from barrier tissues also express CD103 and CD49a (14, 15). In addition, CXCR3 has also been involved with development and homing of T_RM_ cells (16). Increasing evidence has uncovered the roles of T_RM_ cells in the pathogenies of autoimmune disorders, such as psoriasis and rheumatic arthritis (17). Our previous data showed that CD8^+^ T_RM_ cells promoted lupus nephritis (18). Recently, a study showed that the presence of CD4^+^CD69^+^CD103^+^ T_RM_ cells was predictive of disease flares and depletion of T_RM_ cells led to a suppression of colitis activity (19). However, CD103^+^ CD8^+^ T_RM_ cells were shown to be less in inflamed tissue from IBDs compared to non-inflamed biopsies in some reports (20, 21). Therefore, the role of T_RM_ cells in the development of intestinal inflammation behind IBDs and underlying regulation needs further investigation.

Therapeutics targeting immune checkpoints have been promising in the treatment of cancers. Data showed that Programmed cell death protein 1/Programmed deathligand 1 (PD-1/PD-L1) blockade was capable to enhance antitumor response of T_RM_ cells in the tumor microenvironment (22). T-cell immunoreceptor with immunoglobulin and ITIM domains (TIGIT) is a recently identified immune checkpoint receptor that functionally contributes to the impairment of antitumor response (23). TIGIT is a transmembrane glycoprotein that competes with CD226 to bind CD155 (24). TIGIT is expressed exclusively in memory T cells, natural killer (NK) cells, and T_reg_ cells and could have different functions on different cell types (23, 25). Recently, data have revealed that CD226/TIGIT/CD155 axis was involved with human Tfh cell differentiation (26). Adoptive transfer of TIGIT^high^ T_reg_ cells suppressed colitis by inhibiting pro-inflammatory T_H_1 and T_H_17 cell responses specifically (27). In human malignancy, TIGIT has been associated with T-cell exhaustion (28). In addition, TIGIT signaling suppressed CD4^+^ T-cell responses in systemic lupus erythematosus (29). However, little is known about the role of TIGIT in controlling T_RM_ cells in the gut and their contribution to the pathogenesis of IBDs.

Here, we showed that CD4^+^ T_RM_ cells were expanded in the colon of mice with dextran sulfate sodium (DSS)–induced colitis. CD4^+^ T_RM_ cells from mice with DSS-induced colitis were heterogeneous and functionally distinct. CD69^+^CD103^−^ CD4^+^ T_RM_ cells were the major source of interleukin-17A (IL-17A) production in the colon and TIGIT deficiency protected mice from induction of experimental colitis by reducing IL-17A–producing CD69^+^CD103^−^ CD4^+^ T_RM_ cells.

## Methods and Materials

### Animals

TIGIT^−/−^ mice of C57BL/6 background were kindly provided by professor Xingxu Huang from ShanghaiTech University. Mice were bred and maintained in a specific pathogen–free condition at the Experimental Animal Center of Sun Yat-sen University. The study protocol was approved by the Ethics Committee of the Laboratory Animal Center of Sun Yat-sen University and all experiments were performed in accordance with the National Institutes of Health Guide for Care and Use of Animals.

### Colitis Induction

DSS (molecular weight: 36,000–50,000 Da; MP Biomedicals, USA) was added to drinking water at 2% (w/v). Colitis was induced in 8-week-old male littermates of TIGIT^−/−^ or wild-type (WT) mice with 2% DSS *ad libitum* for a consecutive 7 days, followed by water for another 2 days. Mice were monitored daily for weight loss, stool consistency, and hematochezia. Clinical parameters were determined as follows: weight loss (score 0~4: 0 = None; 1 = 1~5%; 2 = 6~10%; 3 = 11~18%; 4 => 18%), stool consistency (score 0~4: 0 = Normal; 1 = Soft but still formed; 2 = Soft; 3 = Very soft, wet; 4 = Watery diarrhea), anal bleeding (score 0~4: 0 = Negative hemoccult; 1 = Negative hemoccult; 2 = Positive hemoccult; 3 = Blood traces in stool visible; 4 = Gross rectal bleeding) (30). Disease activity index was calculated based on weight loss, stool consistency, and anal bleeding. At the end of treatment, mice were anesthetized. The spleen and colon were obtained for subsequent analysis.

### Cell Isolation

Lamina propria mononuclear cells (LPMCs) were isolated as previously described with minor modifications (31). Briefly, colons were cut longitudinally, washed in Hank’s balanced salt solution (HBSS) supplemented with 1% fetal bovine serum (FBS). Tissues were cut into 0.5-cm^2^ pieces and incubated at 37°C for 20 min in HBSS containing 3 mM ethylenediaminetetraacetic acid and 1mM dithiothreitol to remove epithelial cell. After the incubation, epithelial cells in supernatant were discarded by passing through a 100-µm cell strainer. Then, the remaining pieces was minced and incubated with 1× minimum essential medium–α containing 100 U/ml penicillin, 100 μg/ml streptomycin, and 10% FBS, and supplemented with 1 mg/ml collagenase IV (Biosharp, China), 1 mg/ml dispase II (Roche, Basel, Swiss), and 0.5 mg/ml deoxyribonuclease I (Roche, Basel, Swiss) for 60 min in a shaking incubator at 37°C. The resulting cell suspension was filtered through a 70-μm cell strainer (BD, USA) and LPMCs were collected after centrifugation at 300*g* for 10 min.

For splenocyte isolation, mouse spleens were cut into 3- to 5-mm pieces, and smashed using a syringe plunger and then filtered through a 70-μm cell strainer. Red blood cells were removed using red blood cell lysing buffer (Sigma, USA). Prepared single-cell suspensions were further processed flow cytometry analysis.

### Flow Cytometry

For cell surface staining, cells were stained with antibodies against CD45 (Percp/cy5.5, clone#30-F11), CD3 (Alexa Fluor 700, clone#17A2), CD4 (Pacific Blue, clone#GK1.5), CD8 (Alexa Fluor 594, clone#53-6.7), CD69 (APC/Fire 750, clone#H1.2F3), CD103 (PE, clone#2E7), TIGIT (PE/cy7, clone#1G9), CD226 (APC, clone#10E5), CD44 (AF647, clone#IM7), CD62L (BV510, clone#MEL-14), NK1.1 (Pacific Blue, clone#PK136), B220 (AF700, clone#RA3-6B2), CD38 (PE/cy7, clone#90), CD138 (BV421, clone#281-2), CD80 (FITC, clone#16-10A1), and CD11b (BV421, clone#M1/70) at 4°C for 30 min and then washed with phosphate-buffered saline (PBS) twice. For intracellular cytokine staining, cells were first stimulated with 50 ng/ml phorbol 12-myristate 13-acetate, 500 ng/ml ionomycin, and 5 μg/ml brefeldin A (all from Sigma, USA) for 5 h. Cells were then collected and permeabilized with the fixation/permeabilization solution, stained with antibodies against interferon-γ (IFNγ) (APC, clone#XMG1.2), tumor necrosis factor–α (TNFα) (FITC, clone#MP6-XT22), and IL-17A (PE, clone# TC11-18H10.1) at 4°C for 30 min. For Foxp3 and Ki-67 measurement, cells were fixed and permeabilized with a Foxp3 Staining Set (eBioscience, USA) and stained with antibodies against Foxp3 (BV421, clone#MF-14) and Ki-67 (APC, clone#16A8). All the fluorescence-activated cell sorting antibodies were from BioLegend (USA). Flow cytometry was performed on a Cytek™ AURORA. The data were analyzed with FlowJo software (Tree Star). Gating strategy is shown in **Supplementary Figure 1**.

### Histology

For histological analyses, colons were flushed with PBS and cut transversely. Colon tissues were then fixed in 10% neutral buffered formalin and processed for wax embedding. Paraffin sections were hematoxylin and eosin (H&E) stained and subsequently analyzed by bright-field microscopy (Olympus BX63F, Japan). Histologic colitis severity was evaluated as described previously (30). The sum of the two subscores (Tissue damage in DSS-induced colitis, score 0~3; Lamina propria inflammatory cell infiltration in DSS colitis, score 0~3) results in a combined score ranging from 0 (no changes) to 6 (widespread cellular infiltrations and extensive tissue damage).

### Immunohistochemistry

For immunohistochemistry (IHC) analysis, paraffin sections (4 μm) were first dewaxed and rehydrated. Tissue slides were then subjected to antigen-retrieved in a citrate buffer (10 mM sodium citrate, 0.05% Tween 20, pH 6.0) for 15 min. For Ki-67 and CD155 detection, sections were incubated with antibodies against Ki-67 (1:200, Servicebio, China, catalog #GB13030-2) and CD155 (1:300, Abcam, UK, catalog #ab233102) respectively at 4°C overnight. The sections were then washed and subsequent incubated with horseradish peroxidase (HRP)–conjugated Goat anti-Rabbit IgG (1:200, Servicebio, China, catalog #GB23303) for 45 min at room temperature. The subsequent detection was performed using the standard substrate detection of DAB (Servicebio, China, catalog #G1212).

### scRNA-Seq Analysis

Publicly available single-cell RNA-sequencing (scRNA-seq) datasets of DSS-induced colitis were obtained from Gene Expression Omnibus (accession code: GSE148794) (32). The matrix was made up of time-course sequencing data of isolated colon cells from day 0 to day 15 after DSS administration. Cells were included for further analysis if fulfilling all the following criteria: (i) number of expressed genes > 200 and < 3000; (ii) number of total transcripts > 800; and (iii) percentage of mitochondrial genes < 20%. Seurat v4 was utilized to analyze the scRNA-seq data (33). Briefly, highly variable genes were identified based on normalized data firstly. Principle components analysis was performed and top 20 PCs (identified by the elbow plot through JackStraw procedure) were selected for clustering. Non-linear dimensional reduction method, tSNE, was utilized to visualize and explore the data. Next, clusters with strikingly higher expression of CD45 *(Ptprc)*, CD3 delta chain *(Cd3d)*, and T-cell receptor beta-chain constant domains 2 *(Trbc2)* were annotated as T-cell cluster. T-cell cluster was further divided into 10 subclusters by highly differential expressed genes, including CD4 T-1, -2, and -3 cell (*Cd3d* and *Cd4*), CD8 T-1 and -2 cell (*Cd3d* and *Cd8*), natural killer T cell (*Cd3d* and *Klrk1*), natural killer cell (*Klrk1* and *Klrc1*), group 2 innate lymphoid cell (*Gata3*), and Mast cell (*Mcpt1* and *Mcpt2*). Finally, the gene set variation analysis (GSVA) method was used to calculate the enrichment score (ES) of gene signatures and identify differences in expression of gene sets between groups, respectively (34). Based on characteristic genes demonstrated by Zhang et al. (35), ES of tissue residency memory signature (T_RM_ signature score) was calculated for CD4^+^ and CD8^+^ T cells. Gene sets regarding cytokine signaling pathways, including IL-17 and IFNγ, were selected from molecular signatures database (MSigDB) (36) to calculated ES, respectively. The correlation between T_RM_ signature score and ES of cytokine pathways was explored using the Spearman’s correlation test method.

### Statistical Analysis

Data are presented as means ± standard error of the mean (SEM). Statistical analysis regarding scRNA-seq data was performed using R version 4.1.0. Other stastistical analysis were conducted using GraphPad Prism 8.0. The differences were assessed by *t*-test or one-way ANOVA as appropriately followed by adjustment of multiple comparisons. For correlation analysis, Spearman’s correlation coefficient was applied as appropriate. Two-tailed *p* < 0.05 was considered statistically significant.

## Results

### Expansion of CD4^+^ T_RM_ Cells in DSS-Induced Colitis

**Figure 1:**
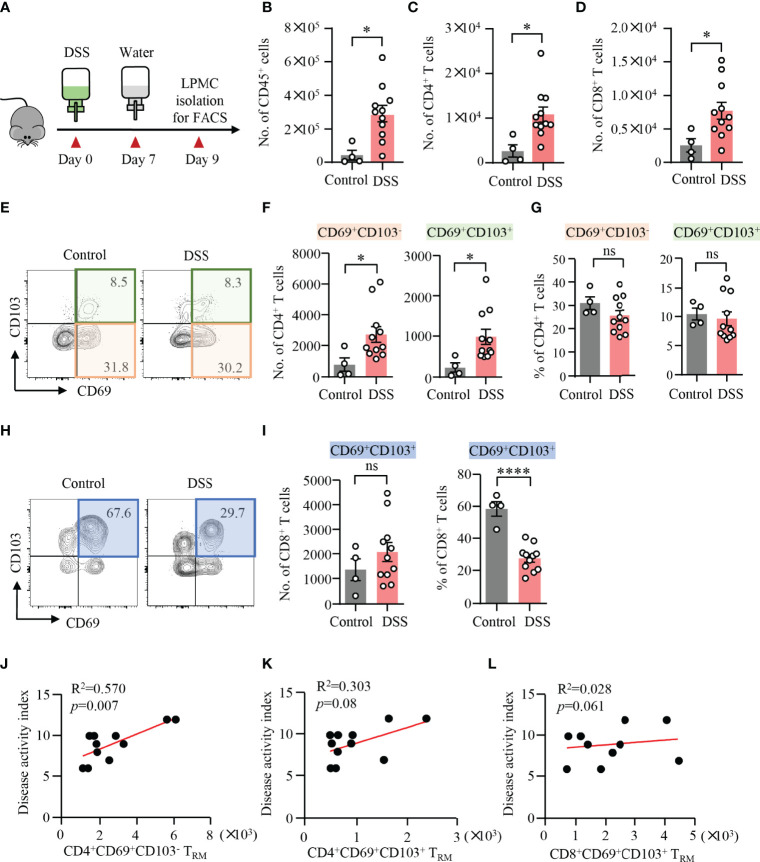
Expansion of CD4^+^ T_RM_ cells in DSS-induced colitis. **(A)** Schematic representation of mouse experiment. Eight-week-old mice were fed with or without DSS for 7 days and switched to water for another 2 days. Disease activities were monitored daily. **(B–L)** Colons were collected from mice with or without DSS-induced colitis. Single cells were prepared and lamina propria mononuclear cells (LPMCs) were isolated. The cell subsets were measured by flow cytometry. **(B)** Numbers of CD45^+^ cells. **(C)** Numbers of CD4^+^ T cells. **(D)** Numbers of CD8^+^ T cells. **(E)** Representative counter plots for CD69 and CD103 expression in CD4^+^ T cells. **(F)** Numbers of CD4^+^CD69^+^CD103^−^ T cells or CD4^+^CD69^+^CD103^+^ T cells measured by flow cytometry. **(G)** Percentages of CD4^+^CD69^+^CD103^−^ T cells or CD4^+^CD69^+^CD103^+^ T cells measured by flow cytometry. **(H)** Representative counter plots for CD69 and CD103 expression in CD8^+^ T cells. **(I)** Numbers and frequency of CD8^+^CD69^+^CD103^+^ T cells measured by flow cytometry. **(J–L)** Correlations between the numbers of CD4^+^CD69^+^CD103^−^ T cells, CD4^+^CD69^+^CD103^+^ T cells, and CD8^+^CD69^+^ CD103^+^ T cells with disease activity index in DSS-induced colitis. Spearman’s R square and a regression line are indicated. n = 4 in the control group and n = 11 in the experiment group. Data are means ± SEM. *p < 0.05, ****p < 0.0001 by Student’s *t*-test. ns, not significant.

DSS-induced colitis was established in WT mice, which was confirmed by weight loss, bloody stool, stool consistency, and pathology (**Figure 1A**). LPMCs were isolated from the colon and measured by flow cytometry. Compared to control mice, the number of CD45^+^ cells, CD4^+^ T cells, and CD8^+^ T cells was significantly increased in mice with DSS-induced colitis (**Figures 1B–D**), suggesting a role of CD4^+^ and CD8^+^ T cells during the development of DSS-induced colitis. It has been shown that the presence of CD4^+^ T_RM_ cells was predictive of disease flares in patients with IBD (19). Based on the presence or the absence of CD103, CD4^+^ T_RM_ cells could be divided into CD4^+^CD69^+^CD103^−^ and CD4^+^CD69^+^CD103^+^ subsets. CD8^+^ T_RM_ cells within mucosal tissues are characterized for the expression of both CD69 and CD103 (11). To investigate the roles of T_RM_ cells in DSS-induced colitis, we further analyzed these cells by flow cytometry. Notably, the numbers of CD4^+^CD69^+^CD103^−^ and CD4^+^CD69^+^CD103^+^ T_RM_ cells were strongly increased in DSS-induced mice when compared to control mice, although the percentages of CD69^+^CD103^−^ and CD69^+^CD103^+^ T_RM_ cells within CD4^+^ T cell were similar (**Figures 1E–G**) indicating that circulating CD4^+^ T cells could differentiate into T_RM_ cells and take residency in the gut during the induction of colitis. In contrast, we observed a decreased percentage of CD8^+^CD69^+^CD103^+^ T_RM_ cells in LPMC from DSS-induced colitis. The number of CD8^+^CD69^+^CD103^+^ T_RM_ cells were similar between DSS-induced mice and control mice (**Figures 1H, I**). These data together suggest a role of CD4^+^ T_RM_ cells in DSS-induced colitis. The decreased percentage of CD8^+^CD69^+^CD103^+^ cells might be a result of the infiltration of circulating CD8^+^ T cells, pointing to the important role of CD4^+^ T_RM_ cells in the pathogenesis of DSS-induced colitis. Phenotypically, most of the CD4^+^CD69^+^CD103^−^, CD4^+^CD69^+^CD103^+^ T_RM_ cells and CD8^+^CD69^+^CD103^+^ T_RM_ cells fell into CD44^+^CD62L^−^ effector memory T cells (T_EM_). T_EM_ cells accounted for nearly 80% of these T_RM_ cells (**Supplementary Figure 2**). Moreover, flow cytometry data showed that the number of CD4^+^CD69^+^CD103^−^ T_RM_ cells were correlated with disease activity index in DSS-induced colitis (**Figure 1J**). However, the number of CD4^+^CD69^+^CD103^+^ and CD8^+^CD69^+^CD103^+^ T_RM_ cells showed no correlation with disease activity index (**Figures 1K,L**). These data suggested a role of CD4^+^CD69^+^ CD103^−^ T_RM_ cells in mediating DSS-induced colitis.

### CD4^+^ T_RM_ Cells in DSS-Induced Colitis Are Heterogeneous

**Figure 2:**
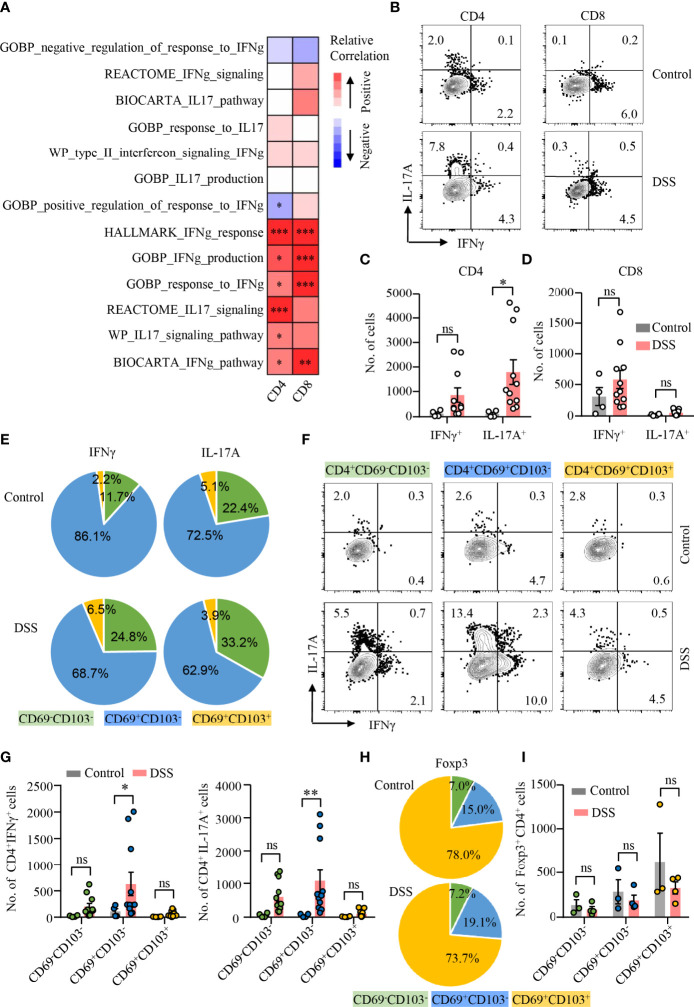
CD4^+^CD69^+^CD103^−^ T_RM_ cells as the major source of IL-17A production during DSS-induced colitis. **(A)** scRNA-seq data derived from GSE148794 were used for analysis. CD4^+^ and CD8^+^ T cells were identified and further adopted to calculate GSVA score. Heatmap showing correlation between T_RM_ signature score of CD4^+^/CD8^+^ T-cell population and ES of indicated gene sets using the Spearman’s correlation analysis. IL-17 and IFNγ signaling pathway gene sets were derived from MSigDB. **(B–I)** DSS-induced colitis was established as described in **Figure 1**. Colons were collected from mice with or without DSS-induced colitis. LPMCs were isolated and intracellular cytokine staining of IFNγ and IL-17A in CD4^+^ and CD8^+^ T cells was performed and measured by flow cytometry. **(B)** Representative counter plots of IFNγ and IL-17A expression in CD4^+^ T cells and CD8^+^ T cells from mice with or without DSS-induced colitis. **(C)** Numbers of IFNγ^+^ and IL-17A^+^ CD4^+^ T cells. **(D)** Numbers of IFNγ^+^ and IL-17A^+^ CD8^+^ T cell. **(E)** Distribution of IFNγ^+^ and IL-17A^+^ cells among CD4^+^CD69^−^CD103^−^, CD4^+^CD69^+^CD103^−^, and CD4^+^CD69^+^ CD103^+^ subsets from mice with or without DSS-induced colitis. **(F)** Representative counter plots of IFNγ and IL-17A expression in CD4^+^CD69^−^CD103^−^, CD4^+^CD69^+^CD103^−^, or CD4^+^CD69^+^CD103^+^ subsets. **(G)** Numbers of IFNγ^+^ and IL-17A^+^ CD4^+^ T cells in mice with DSS-induced colitis to control mice. **(H)** FoxP3 expression in CD4^+^ T cells was measured by flow cytometry. Distribution of CD4^+^CD69^−^CD103^−^, CD4^+^CD69^+^CD103^−^, and CD4^+^CD69^+^CD103^+^ subsets in FoxP3^+^ cells from mice with or without DSS-induced colitis was shown. **(I)** Numbers of FoxP3^+^ CD4^+^ T cells in mice with DSS-induced colitis and control mice were summarized. Each dot represents an independent mouse sample. Data are means ± SEM. *p < 0.05, **p < 0.01, ***p < 0.001 by Student’s *t*-test. ns, not significant.

CD4^+^ T cells are heterogeneous and can be classified into several distinct subsets based on their expression of certain transcription factors and cytokines (37). To investigate the pathological role of T_RM_ cells in colitis, scRNA-seq datasets of colonic cells from DSS-induced colitis were obtained publicly. Dimensional reduction analysis of 13566 single colonic cell identified 11 clusters (**Supplementary Figure 3A**). According to the scRNA-seq data, T-cell cluster was segregated based on increased expression of T-cell marker (*Ptprc*, *Cd3d*, and *Trbc2*) (**Supplementary Figure 3B**). T-cell cluster was further divided into 10 subclusters (**Supplementary Figures 3C, D**). GSVA technique allowed sensitive identification for enrichment of T_RM_ gene signature and signaling pathways between different groups, respectively. Spearman’s correlation analysis demonstrated that IL-17 and IFNγ pathways were both correlated with CD4^+^ T_RM_ cell signature score positively, whereas IFNγ pathways were significantly associated with CD8^+^ T_RM_ cell signature score (**Figure 2A**).

To further uncover the functional heterogeneity of CD4^+^ T_RM_ cells, we first measured IL-17A and IFNγ expression in CD4^+^ and CD8^+^ T cells in LPMC by flow cytometry (**Figure 2B**). Percentages of IL-17A– or IFNγ-expressing CD4^+^ T cells were increased, whereas percentages of IL-17A– or IFNγ-expressing CD8^+^ T cells were not changed in mice with colitis compared with control mice (**Supplementary Figure 4A**). Notably, the number of CD4^+^ T cells expressing IL-17A was significantly increased in mice with DSS-induced colitis, while numbers of CD4^+^ T cells expressing IFNγ remained unchanged (**Figure 2C**). In contrast, the numbers as well as percentages of IFNγ or IL-17A–expressing CD8^+^ T cells were not changed in mice with colitis. (**Figure 2D**; **Supplementary Figure 4A**). We then analyzed the composition of IL-17A– or IFNγ-expressing CD4^+^ T cell. Surprisingly, CD69^+^CD103^−^ CD4^+^ T_RM_ cells were the major source of IL-17A and IFNγ in the gut of mice with or without DSS-induced colitis. CD69^+^CD103^−^ CD4^+^ T_RM_ cells accounted for 62.9% and 72.5% of IL-17A^+^ CD4^+^ T cells in the colon of DSS-induced colitis or control mice, representing the major source of IL-17A production. In contrast, CD69^+^CD103^+^ CD4^+^ T_RM_ cells only accounted for 2.2% of IFNγ- and 5.1% of IL-17A–expressing CD4^+^ T cells in control mice, 6.5% of IFNγ- and 3.9% of IL-17A–expressing CD4^+^ T cells in DSS-induced colitis, respectively. CD69^−^CD103^−^ CD4^+^ T cells accounted for 11.7% of IFNγ-expressing CD4^+^ T cells in control mice and 24.8% in DSS-induced colitis. Among IL-17A^+^ CD4^+^ T cells, CD69^−^CD103^−^ CD4^+^ T cells accounted for 22.4% in control mice and 33.2% in DSS-induced colitis. (**Figure 2E**). Albeit IFNγ expression was increased in CD69^−^CD103^−^ and CD69^+^CD103^+^ CD4^+^ T-cell subsets during DSS-induced colitis, only small percentages of CD69^−^CD103^−^ CD4^+^ T cells and CD69^+^CD103^+^ CD4^+^ T_RM_ cells expressed IFNγ (**Figure 2F**; **Supplementary Figure 4B**). IFNγ expression was not different in CD69^+^CD103^−^ subset between mice with or without DSS-induced colitis (**Figure 2F**; **Supplementary Figure 4B**). Notably, IL-17A expression was largely increased in all the three subsets of CD4^+^ T cells during DSS-induced colitis. The numbers of CD69^−^CD103^−^ T cells and CD69^+^CD103^+^ CD4^+^ T_RM_ cells expressing IFNγ or IL-17A were similar between mice with or without colitis (**Figure 2G**). CD69^+^CD103^−^ CD4^+^ T_RM_ cells only accounted for 19.1% and 15.0% of Foxp3^+^ T_reg_ cells in the colon of mice with or without DSS-induced colitis, respectively. Majority of the CD69^+^CD103^+^ CD4^+^ T_RM_ cells were Foxp3^+^ T_reg_ cells that more than 70% of Foxp3^+^ T_reg_ cells were CD69^+^CD103^+^ CD4^+^ T_RM_ cells both in mice with or without colitis (**Figure 2H**). However, neither percentages nor cell numbers of Foxp3^+^ T_reg_ cells were changed in DSS-induced colitis (**Figure 2I**; **Supplementary Figure 4C**). These data revealed the heterogeneity of CD4^+^ T_RM_ cells functionally and suggested a role of IL-17A–expressing CD69^+^CD103^−^ CD4^+^ T_RM_ cells in DSS-induced colitis.

### TIGIT Expression in CD4^+^ T Cells Is Increased During DSS-Induced Colitis

**Figure 3:**
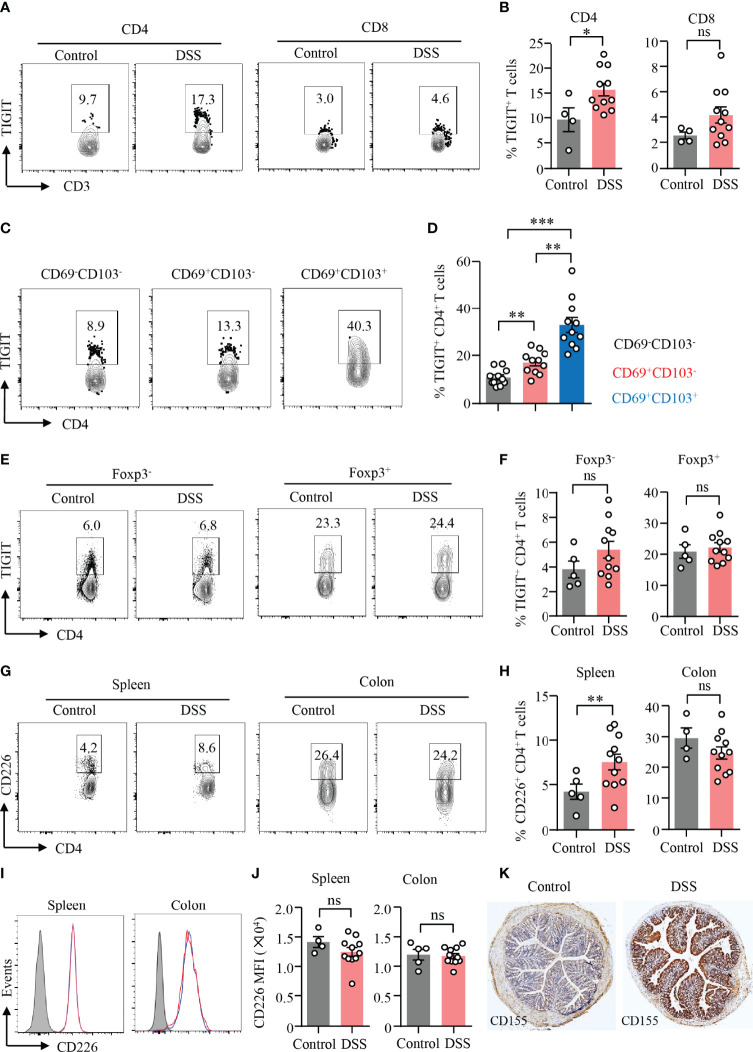
TIGIT expression in CD4^+^ T cells was increased during DSS-induced colitis. DSS-induced colitis was established as described in **Figure 1**. Single-cell suspension of the spleen or LPMC was prepared as described in **Figure 1**. **(A)** TIGIT expression in CD4^+^ and CD8^+^ T cells from LPMC was measured by flow cytometry. Representative counter plots were shown. **(B)** Frequencies of TIGIT^+^ CD4^+^ T cells and TIGIT^+^ CD8^+^ T cells from LPL were summarized. **(C)** Representative counter plots for TIGIT expression in CD4^+^CD69^−^CD103^−^ T cells, CD4^+^CD69^+^CD103^−^ T cells, or CD4^+^CD69^+^CD103^+^ T cells. **(D)** Frequencies of TIGIT^+^ cells among CD4^+^CD69^−^CD103^−^ T cells, CD4^+^CD69^+^CD103^−^ T cells, or CD4^+^ CD69^+^ CD103^+^ T-cell subsets in DSS-induced colitis. **(E)** TIGIT expression in CD4^+^FoxP3^+^ and CD4^+^FoxP3^−^ T cells in the spleen was measured by flow cytometry. Representative counter plots were shown. **(F)** Frequencies of TIGIT^+^ Foxp3^+^CD4^+^ T cells and Foxp3^−^CD4^+^ T cells were summarized. **(G–J)** CD226 expression in CD4^+^ and CD8^+^ T cells from LPMC or spleen of mice with or without DSS-induced colitis was measured by flow cytometry. **(G)** Representative counter plots of CD226 expression in CD4^+^ T cells from the spleen or LPMC of mice with or without DSS-induced colitis. **(H)** Frequencies of CD226^+^ CD4^+^ T cells were summarized. **(I)** Representative histograms of CD226 expression in CD8^+^ T cells from the spleen or LPMC of mice with or without DSS-induced colitis. **(J)** Mean fluorescence intensity (MFI) of CD226 expression in CD8^+^ T cells. **(K)** Colon sections from mice with or without DSS-induced colitis were stained with antibody against CD155. Representative images of CD155 staining by immunohistochemistry were shown. Original magnification: ×4. Data are means ± SEM. *p < 0.05, **p < 0.01, ***p < 0.001, by Student’s *t*-test. ns, not significant.

TIGIT is an emerging immune checkpoint that is expressed in T cells and NK cells mostly. It has been shown that TIGIT suppresses immune cell responses against cancer (23). TIGIT expression in T cells or NK cells was at low level except that in T_reg_ cells (**Supplementary Figure 5**). To investigate the role of TIGIT in gut inflammation, an experimental model of DSS-induced colitis was adopted in this study. We found that the expression of TIGIT was strongly increased in CD4^+^ T cells in the inflamed gut (**Figures 3A, B**). Although there was an increasing trend for the expression of TIGIT in CD8^+^ T cells, the difference was not significant (**Figures 3A, B**). We next analyzed TIGIT expression among subsets of CD4^+^ T cells from DSS-induced colitis by flow cytometry. The data revealed that TIGIT expression was relatively low in CD69^−^CD103^−^ cells, whereas CD69^+^CD103^+^ subset showed the highest percentage of TIGIT expression among subsets of CD4^+^ T cells (**Figures 3C, D**). Although CD69^+^CD103^+^ cells accounted for the majority of T_reg_ cells in both colons, TIGIT expression in T_reg_ cells was not different in mice with or without colitis (**Figures 3E, F**). TIGIT expression was similar in CD4^+^ T cells from the spleen of colitis or control mice (**Figures 3E, F**). TIGIT competes with the costimulatory receptor CD226 for binding to CD155 (25). Flow cytometry analysis showed that CD226 expression was increased in CD4^+^ T cells from spleens in DSS-induced colitis. Although CD226 expression was significantly higher in CD4^+^ T cells from the colon than that from the spleen, CD226 expression was no changed in CD4^+^ T cells from the colon of mice with DSS-induced colitis (**Figures 3G, H**). Of note, TIGIT expression was not different in CD8^+^ T cells neither from the spleen nor from colon (**Figures 3I, J**). These data suggested that CD226 might not be involved with T-cell activation during DSS-induced colitis. Moreover, CD155 expression in the colon was measured by IHC. CD155 was detected in the non-inflamed colon but at low level. Notably, CD155 expression in the colon was robustly induced in DSS-induced colitis (**Figure 3K**), indicating a role of CD155 in the pathogenesis of DSS-induced colitis.

### TIGIT Deficiency Neither Affect T-Cell Residency Nor Induce Spontaneous Autoimmune Response in the Gut

**Figure 4:**
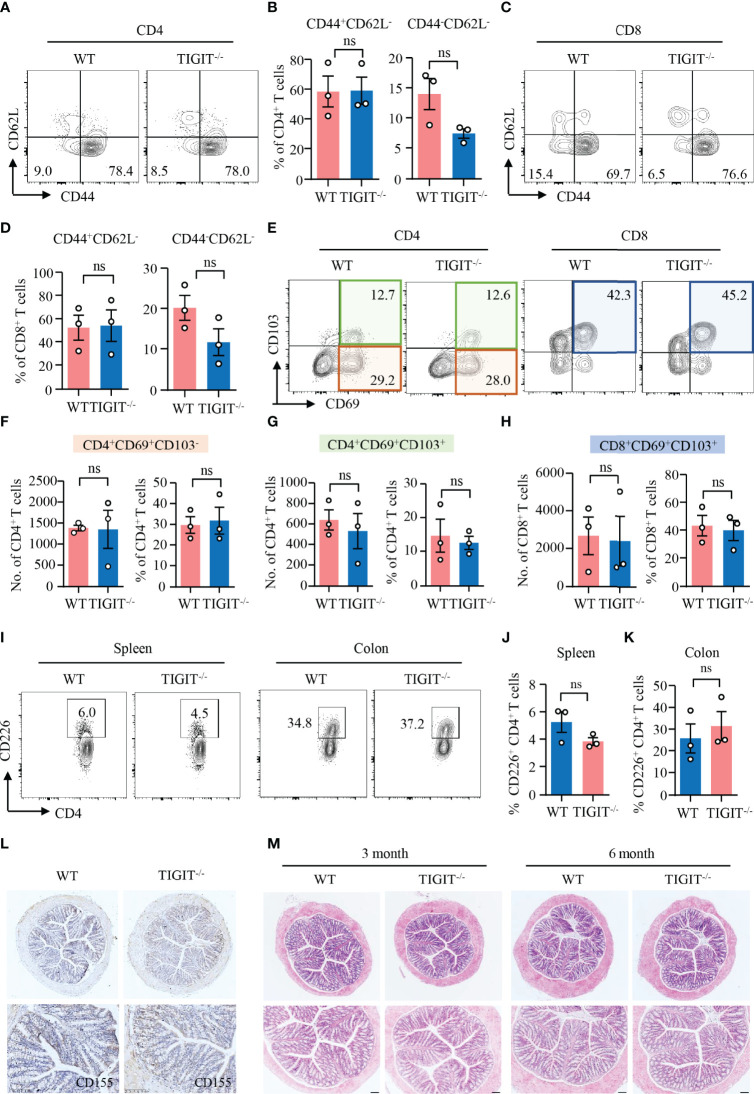
TIGIT deficiency neither affected T-cell residency nor induced spontaneous autoimmune response in the gut. Colons were collected from TIGIT^−/−^ or WT mice of 8 weeks old and LPMC were isolated for flow cytometry. **(A–D)** CD44 and CD62L expression in CD4^+^ and CD8^+^ T cells was measured by flow cytometry. Representative counter plots were shown. The percentages of CD44^+^CD62^−^ or CD44^−^CD62L^−^ in CD4^+^ and CD8^+^ T cells were summarized from three independent mice. **(E–H)** CD103 and CD69 expression in CD4^+^ or CD8^+^ T cells from LPMC was measured by flow cytometry. Representative counter plots were shown and data were from three independent mice. **(I)** Representative counter plots of CD226 expression in CD4^+^ T cells from the spleen or LPMC. **(J, K)** Frequencies of CD226^+^ CD4^+^ T cells from spleen or colon were summarized. **(L)** CD155 expression in the colon was measured by immunohistochemistry. Representative images were shown. Original magnification: ×10. **(M)** Colons from WT and TIGIT^−/−^ mice embedded with paraffin were sectioned and stained with H&E. Representative images were shown. Original magnification: ×4 (top), ×10 (bottom). ns, not significant.

Data above revealed that TIGIT expression was relatively high in CD69^+^CD103^−^ and CD69^+^CD103^+^ T_RM_ cells. Here, we were to investigate whether TIGIT has the impacts on T-cell residency in the colon. CD62L is a homing receptor that mediates the entry of naïve T cells to peripheral lymph nodes that CD62L expression is low in T_RM_ cells (38). Flow cytometry analysis revealed that most of the CD4^+^ and CD8^+^ T cells from the colons of TIGIT^−/−^ or WT mice without colitis did not express CD62L (**Figures 4A–D**). CD44, as an activation marker of T cells, whose expression in CD4^+^ and CD8^+^ T cells from the colons was not changed in TIGIT^−/−^ mice when compared to that from WT mice (**Figures 4A–D**). Moreover, the numbers and percentages of CD4^+^CD69^+^CD103^−^, CD4^+^CD69^+^CD103^+^ and CD8^+^CD69^+^CD103^+^ T_RM_ cells were very similar between TIGIT^−/−^ and WT mice (**Figures 4E-H**). The composition and functional status of immune cells in the spleen were similar between TIGIT^−/−^ and WT mice (**Supplementary Figures 6**, **7**). These data suggested that TIGIT deficiency was not involved with autoimmune response in the gut.

Next, we investigated the impacts of TIGIT deficiency on CD226 and CD155 expression by flow cytometry and IHC, respectively. The flow cytometry data showed that CD226 expression in CD4^+^ T cells from the spleen or colon of TIGIT^−/−^ mice was similar compared with WT mice (**Figures 4I–K**). Moreover, CD155 expression in the colon was not affected when TIGIT was knocked out as measured by IHC (**Figure 4L**). Pathologically, we did not observe changes in the colon between TIGIT^−/−^ and WT mice. Epithelial layers of colon were intact and immune infiltration was absent in both TIGIT^−/−^ and WT mice (**Figure 4M**). These data suggested that TIGIT exerted no effects on T-cell residency and TIGIT deficiency was not sufficient to induce spontaneous autoimmune response in the gut.

### TIGIT Deficiency Reduces IL-17A–Producing CD69^+^CD103^−^ CD4^+^ T_RM_ Cells in DSS-Induced Colitis

**Figure 5:**
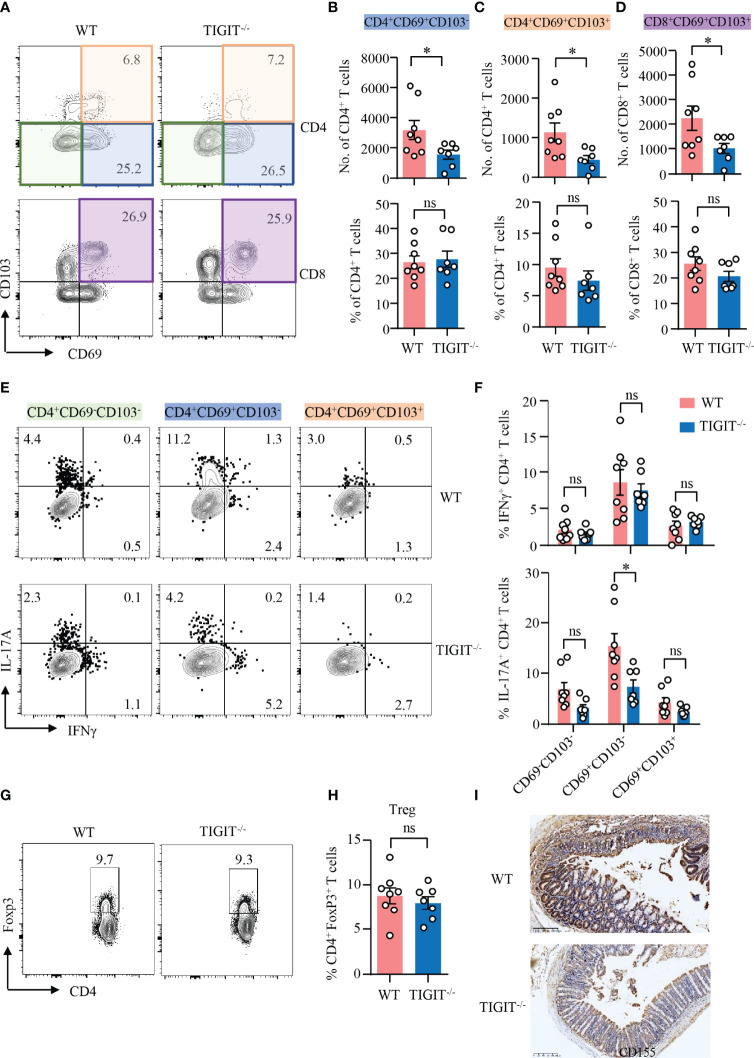
TIGIT deficiency reduced IL-17A–producing CD4^+^CD69^+^CD103^−^ T cells in DSS-induced colitis. DSS-induced colitis was established as in **Figure 1**. LPMCs were isolated from colon of TIGIT^−/−^ or WT mice with DSS-induced colitis. **(A–D)** CD69 and CD103 expression in CD4^+^ and CD8^+^ T cells in LPMC from TIGIT^−/−^ or WT mice was measured by flow cytometry. Representative counter plots for CD69 and CD103 expression were shown. **(B)** Numbers and frequencies of CD4^+^CD69^+^CD103^−^ T cells. **(C)** Numbers and frequencies of CD4^+^CD69^+^CD103^+^ T cells. **(D)** Numbers and frequencies of CD8^+^CD69^+^ CD103^+^ T cells. **(E)** IFNγ and IL-17A expression in CD4^+^ T cells from LPL was measured by flow cytometry. Representative flow cytometry of IFNγ and IL-17A expression of CD4^+^CD69^−^CD103^−^ T cells, CD4^+^CD69^+^ CD103^−^ T cells, or CD4^+^CD69^+^CD103^+^ T cells. **(F)** Frequencies of IFNγ^+^ and IL-17A^+^ T cells among CD4^+^CD69^−^CD103^−^ T cells, CD4^+^CD69^+^CD103^−^ T cells, or CD4^+^CD69^+^CD103^+^ T cells. **(G, H)** FoxP3 expression in CD4^+^ T cells from spleens of TIGIT^−/−^ or WT mice was measured by flow cytometry. Percentages of FoxP3^+^ CD4^+^ T cells were summarized and representative counter plots were shown. **(I)** Colon sections from TIGIT^−/−^ or WT mice with DSS-induced colitis were stained with antibody against CD155 and visualized using HRP-conjugated secondary antibody. Representative images of CD155 staining by immunohistochemistry were shown. Original magnification: ×10. Data are means ± SEM. WT = 8, TIGIT^−/−^ = 7. *p < 0.05 by Student’s *t*-test. ns, not significant.

Data above showed that TIGIT deficiency showed no effect on T-cell residency in the gut. We further investigated the role of TIGIT in T-cell residency during DSS-induced colitis. TIGIT^−/−^ mice showed decreased numbers of CD69^+^CD103^−^ and CD69^+^CD103^+^ CD4^+^ T_RM_ cells, as well as CD69^+^CD103^+^ CD8^+^ T_RM_ cells in the colons. However, the percentages of these cells in the colons of TIGIT^−/−^ mice were not changed compared with WT mice (**Figures 5A–D**), indicating that the reduced cell numbers might due to the overall ameliorated infiltration. To further study the functional connection between TIGIT and CD4^+^ T_RM_ cells, intracellular cytokine production was measured in CD4^+^ T cells from the colons of TIGIT^−/−^ or WT mice by flow cytometry. Similar to WT mice, IFNγ expression was not changed in CD69^−^CD103^−^, CD69^+^CD103^−^ and CD69^+^CD103^+^ CD4^+^ T cells from TIGIT^−/−^ mice. Interestingly, IL-17A expression was downregulated in CD69^+^CD103^−^ CD4^+^ T cells from TIGIT^−/−^ mice when compared to that from WT mice specifically. IL-17A expression in CD69^−^CD103^−^ and CD69^+^CD103^+^ CD4^+^ T cells were otherwise not changed in TIGIT^−/−^ mice (**Figures 5E, F**). The percentages of Foxp3^+^ T_reg_ cells were similar between TIGIT^−/−^ and WT mice (**Figures 5G, H**). In terms of the phenotype of CD4^+^ T cells from the spleen, percentages of CD4^+^ T cells in immune cells and CD4^+^ T_N_ in all CD4^+^ T cells were increased in TIGIT^−/−^ mice, while CD8^+^ T_N_ and T_CM_ were also increased. The percentages of CD44^+^CD4^+^ and CD44^+^CD8^+^ T cells in the gut were decreased in TIGIT^−/−^ mice. The percentage of Ki-67^+^ CD4^+^ T cells was similar, whereas percentage of Ki-67^+^ CD8^+^ T cells was increased in TIGIT^−/−^ mice (**Supplementary Figure 8**). To investigate whether TIGIT deficiency affects CD155 expression in the colon, CD155 expression was detected by IHC, which revealed that CD155 expression in TIGIT^−/−^ mice was reduced during DSS-induced colitis (**Figure 5I**).

### TIGIT Deficiency Protects Mice From DSS-Induced Colitis

**Figure 6:**
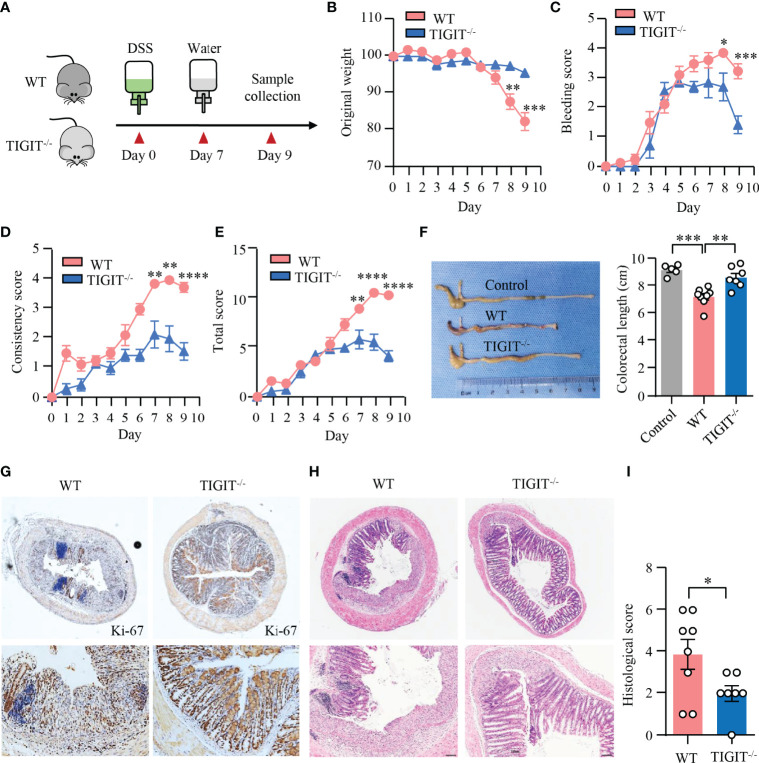
TIGIT deficiency protected mice from DSS-induced colitis. **(A)** Schematic representation of experiment design. Male littermates of TIGIT^−/−^ or WT mice were fed with DSS to induce colitis. Loss of body weight **(B)**, bleeding score **(C)**, stool consistency score **(D)**, and total score of disease activity index **(E)** were measured daily. **(F)** Colon length was measured at the end of the experiment and representative images of the colons from TIGIT^−/−^ or WT mice with DSS-induced colitis or control mice without DSS-induced colitis were shown. **(G)** Colon sections were stained with antibody against Ki-67 and visualized using HRP-conjugated secondary antibody. Representative images of Ki-67 staining in colonic tissues from TIGIT^−/−^ or WT mice with DSS-induced colitis were shown. Original magnification: ×4 (top), 20× (bottom). **(H, I)** Representative H&E images of the colon **(H)** and histological scores **(I)**. Original magnification: ×4 (top), ×10 (bottom). WT (n = 8) or TIGIT^−/−^ mice (n = 7). Data are means ± SEM. *p < 0.05, **p < 0.05, ***p < 0.001, ****p < 0.0001 by Student’s *t*-test.

To assess the impact of TIGIT deficiency on DSS-induced colitis, colitis was induced in TIGIT^−/−^ and WT mice as in **Figure 1** (**Figure 6A**). Notably, TIGIT^−/−^ mice were clinically protected from DSS-induced injury in terms of weight loss (**Figure 6B**). In addition, scores for intestinal bleeding and stool consistency were significantly lower in TIGIT^−/−^ mice (**Figures 6C, D**). As a result, TIGIT^−/−^ mice showed lower disease activity index (**Figure 6E**) and longer colon length (**Figure 6F**) compared with WT mice. Furthermore, IHC of colons showed that the number of Ki-67^+^ epithelial cells was profoundly increased in TIGIT^−/−^ mice (**Figure 6G**), indicating better recovery of the epithelial barrier. TIGIT^−/−^ mice showed reduced lamina propria inflammatory cell infiltration and epithelial injury profoundly when compared with WT mice (**Figures 6H, I**). These data demonstrate that TIGIT deficiency protects mice from DSS-induced colitis.

## Discussion

IBD is characterized by immune-mediated intestinal inflammation that is driven by both genetic predisposition and environmental factors. An abnormal immune response to gut microbiotas plays an important role in the pathogenesis of IBD (2, 39). However, the pathogenesis of IBD is far from understood. Most IBD therapies target inflammatory pathways involved in the dysfunctional immune response. Biologic agents targeting TNF signaling are some of the most effective agents in the care of IBD (40). Here, our data uncover a pathogenic role of CD69^+^CD103^−^ CD4^+^ T_RM_ cells in DSS-induced colitis. CD69^+^CD103^−^ CD4^+^ T_RM_ cells are the major source of IL-17A production in the colon of mice with DSS-induced colitis. TIGIT expression is strongly increased in colonic CD4^+^ T cells from mice with DSS-induced colitis. TIGIT deficiency impairs IL-17A production in CD69^+^CD103^−^ CD4^+^ T_RM_ cells specifically, resulting in lower disease activity index and reduced pathological injury in mice with DSS-induced colitis. Together, we provide evidence that TIGIT might serve as a pathogenic factor in DSS-induced colitis.

T_RM_ cells represent a subset of long-lived memory T cells that occupy epithelial and mucosal tissues without recirculating (41). The rise of T_RM_ cells in the local microenvironment could be the results of T_RM_ cell expansion locally or the transformation of circulating T cells into T_RM_ cells (42, 43). The existence of CD4^+^ and CD8^+^ T_RM_ cells at barrier surfaces has been demonstrated previously (44, 45). T_RM_ cells have been involved with several types of autoimmune skin diseases including vitiligo, psoriasis, cutaneous lupus erythematosus, and alopecia (46). As for colitis, it has been shown that T_RM_ cells are increased in the gut of patients with IBD and have a pro-inflammatory phenotype that CD69^+^ T cells expressed higher levels of *IFNγ*, *IL-13*, *IL-17A*, and *TNF* mRNA than CD69^−^ T cells (19). Data have shown that CD4^+^ T_RM_ cells can be divided into CD69^+^CD103^−^ and CD69^+^CD103^+^ subsets (11). Our data confirmed that the numbers of both CD69^+^CD103^−^ and CD69^+^CD103^+^ CD4^+^ T_RM_ cells were significantly increased in the colon of DSS-induced colitis, which was associated with more severe disease activities, pointing to the critical role of T_RM_ cells in the development of colitis. Although IFNγ-producing CD8^+^ T_RM_ cells are a pathological hallmark of immune checkpoint inhibitor treatment related colitis (47), we did not observe the expansion and activation of CD8^+^ T_RM_ cells in DSS-induced colitis.

Although CD4^+^ T_RM_ cells has been associated with flares in IBD, functional analysis of the heterogeneity of CD4^+^ T_RM_ cells has not been studied. Here, IL-17A and IFNγ production was strongly increased in CD4^+^ T cells from mice with colitis. Cytokine analysis revealed that CD69^+^CD103^−^ CD4^+^ T_RM_ cells were the major source of IL-17A and IFNγ production in the colon, whereas CD69^+^CD103^+^ CD4^+^ T_RM_ cells were predominantly Foxp3^+^ T_reg_ cells. CD4^+^ T cell–derived cytokines such as IL-17A and IFNγ are pro-inflammatory and have been shown to be pathogenic in murine models and in patients with IBD (5), whereas T_reg_ cells protect mice from DSS-induced colitis (48). During the induction of colitis, IL-17A expression was notably increased in CD69^+^CD103^−^ CD4^+^ T_RM_ cells. IFNγ expression was however not changed. In addition, the percentages of T_reg_ cells were similar between mice with or without DSS-induced colitis. For the first time, our data revealed the heterogeneity of CD4^+^ T_RM_ cells in the colon of mice with DSS-induced colitis. CD69^+^CD103^−^ CD4^+^ T_RM_ cells could play an important role in controlling DSS-induced colitis by producing IL-17A.

TIGIT, as an immune checkpoint molecule expressed on memory T cells, NK cells, and T_reg_ cells, suppresses T-cell activation by promoting the generation of mature immunoregulatory dendritic cells (24). TIGIT competes with CD226 for binding to their common ligand CD155. However, TIGIT exhibits higher binding priority than CD226 (24). TIGIT inhibits CD8^+^ T-cell proliferation and activation by downregulating TCR expression directly (49) or reduces TCR-induced p-ERK signaling (50). In models of cancers and chronic viral infection, blockade of TIGIT was correlated with enhanced CD8^+^ T-cell effector functions (51–53). TIGIT promoted CD8^+^ T-cell exhaustion in colorectal cancer (54) and impaired antigen-specific T cells in melanoma (55). In addition, binding of TIGIT on NK cells to CD155 suppresses NK cell–mediated cytotoxicity and IFNγ production (56, 57). TIGIT in CD4^+^ T cells is far less understood. Here, our data revealed TIGIT expression in CD4^+^ T cells from LPMC was notably increased in mice with DSS-induced colitis. Concurrently, we also detected the induction of CD155 in the colon during DSS-induced colitis. However, CD226 expression in CD4^+^ and CD8^+^ T cells was not changed. These data suggested that TIGIT/CD155 interaction could play a role during the development of DSS-induced colitis. TIGIT deficiency led to decreased numbers of CD69^+^CD103^−^ and CD69^+^CD103^+^ CD4^+^ T_RM_ cells in the colon during DSS-induced colitis. The percentages of these cells were however not changed. Moreover, the percentages of CD4^+^ and CD8^+^ T_RM_ cells in the LPMC were similar between TIGIT^−/−^ and WT mice without colitis, indicating that TIGIT has no effects on T-cell residency in the gut. Albeit data have shown that TIGIT functioned as an immune checkpoint in several autoimmune disorders (58), we did not observe T-cell activation and spontaneous autoimmune response in the gut of TIGIT^−/−^ mice before the induction of colitis.

Our further data revealed that TIGIT expression was notably increased in CD4^+^ T cells from DSS-induced colitis. IL-17A expression in CD69^+^CD103^−^ CD4^+^ T_RM_ cells was impaired in TIGIT^−/−^ mice during the induction of colitis. However, IFNγ expression in T cells or IL-17A expression in CD69^+^CD103^+^ and CD69^−^CD103^−^ was similar between TIGIT^−/−^ and WT mice. Here, we showed that TIGIT was required for IL-17A production by CD69^+^CD103^−^ CD4^+^ T_RM_ cells during DSS-induced colitis. Albeit it has been shown that TIGIT had T-cell–intrinsic inhibitory functions (49), the decreased expression of CD155 in the colon of TIGIT^−/−^ mice could lead to reduced TIGIT/CD155 interaction, which might contribute to decreased IL-17A production during DSS-induced colitis in the current study. In addition, a study has also shown that TIGIT enhanced T_H_2 immunity in mice with experimental allergic disease through interaction with CD155 expressed in dendritic cell (59). Thus, the function of TIGIT on CD4^+^ T cells could be different due to the intrinsic diverseness of disease model as well as ligand expression in the microenvironment.

It has been shown that TIGIT-Ig postponed onset of proteinuria and reduced serum concentrations of autoantibodies in murine lupus (60). Overexpression of TIGIT suppressed CD4^+^ T cells and ameliorated the severity of rheumatoid arthritis in mouse models (61). TIGIT^−/−^ mice were more susceptible to the induction of experimental autoimmune encephalomyelitis (49). Here, our data revealed that TIGIT^−/−^ mice were resistant to DSS-induced colitis when compared with WT mice. TIGIT^−/−^ mice were clinically protected from DSS-induced injury in terms of weight loss, disease activity, colon length, and pathological change. A hallmark of IBD is the alteration of microbiota composition in the gut (62). Accumulating evidence suggests that IBDs result from an inappropriate immune response to intestinal microbes in a genetically susceptible host (63), which is different from other autoimmune diseases with immune response to autoantigens (64). It has also been shown that TIGIT blockade decreased T-cell function during sepsis (65). Thus, the role of TIGIT in inflammatory disease appears to vary and may be context-dependent. Nevertheless, further studies are needed to investigate the roles of TIGIT in the pathogenesis of DSS-induced colitis and the underlying mechanisms.

Together, we provide evidence that CD4^+^ T_RM_ cells are heterogeneous and functionally distinct regarding different subsets in DSS-induced colitis. CD69^+^CD103^−^ CD4^+^ T_RM_ cells are the major source of IL-17A production in the gut of mice with DSS-induced colitis. TIGIT deficiency protects mice from DSS-induced colitis by reducing IL-17A–producing CD69^+^CD103^−^ CD4^+^ T_RM_ cells. Together, this study reveals a novel mechanism for the regulation of gut inflammation and might have potential therapeutic value in the treatment of IBDs.

## Data Availability Statement

The original contributions presented in the study are included in the article/**Supplementary Material**, further inquiries can be directed to the corresponding author/s.

## Ethics Statement

The study was approved by the Ethics Committee of the Laboratory Animal Center of Sun Yat-sen University.

## Author Contributions

HZ conceived the study. ML maintained TIGIT^−/−^ mice. BC, BY, ML, JL, SW, and YL performed experiments. BC, HZ, NY, and ZK analyzed and interpreted the data. HZ and BC wrote the manuscript with all authors providing feedback. All authors contributed to the article and approved the submitted version.

## Funding

This work is supported by the National Natural Science Foundation of China 81971519, 81671593, 81471598 to N.Y. and National Natural Science Foundation of China 82071819 to H.Z.

## Conflict of Interest

The authors declare that the research was conducted in the absence of any commercial or financial relationships that could be construed as a potential conflict of interest.

## Publisher’s Note

All claims expressed in this article are solely those of the authors and do not necessarily represent those of their affiliated organizations, or those of the publisher, the editors and the reviewers. Any product that may be evaluated in this article, or claim that may be made by its manufacturer, is not guaranteed or endorsed by the publisher.
