# Supplementary material for: TIGIT Deficiency Protects Mice From DSS-Induced Colitis by Regulating IL-17A–Producing CD4+ Tissue-Resident Memory T Cells
Source: Front Immunol. 2022 Jul 1;13:931761. doi: 10.3389/fimmu.2022.931761 (PMC9283574; doi:10.3389/fimmu.2022.931761)
Supplement: Supplementary file 1 [file DataSheet_1.docx]

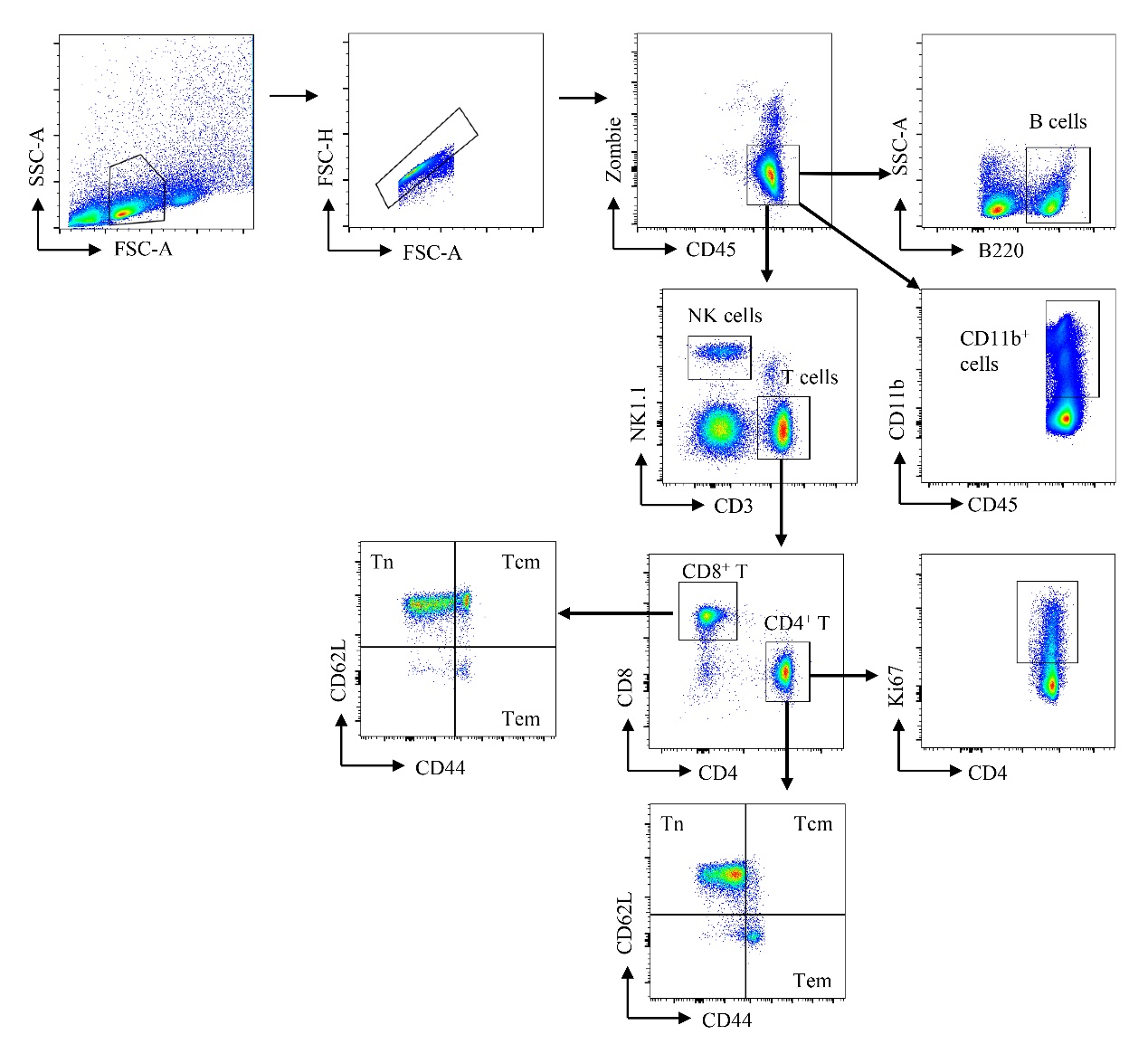


**Supplementary Figure 1**. **Gating strategy.**


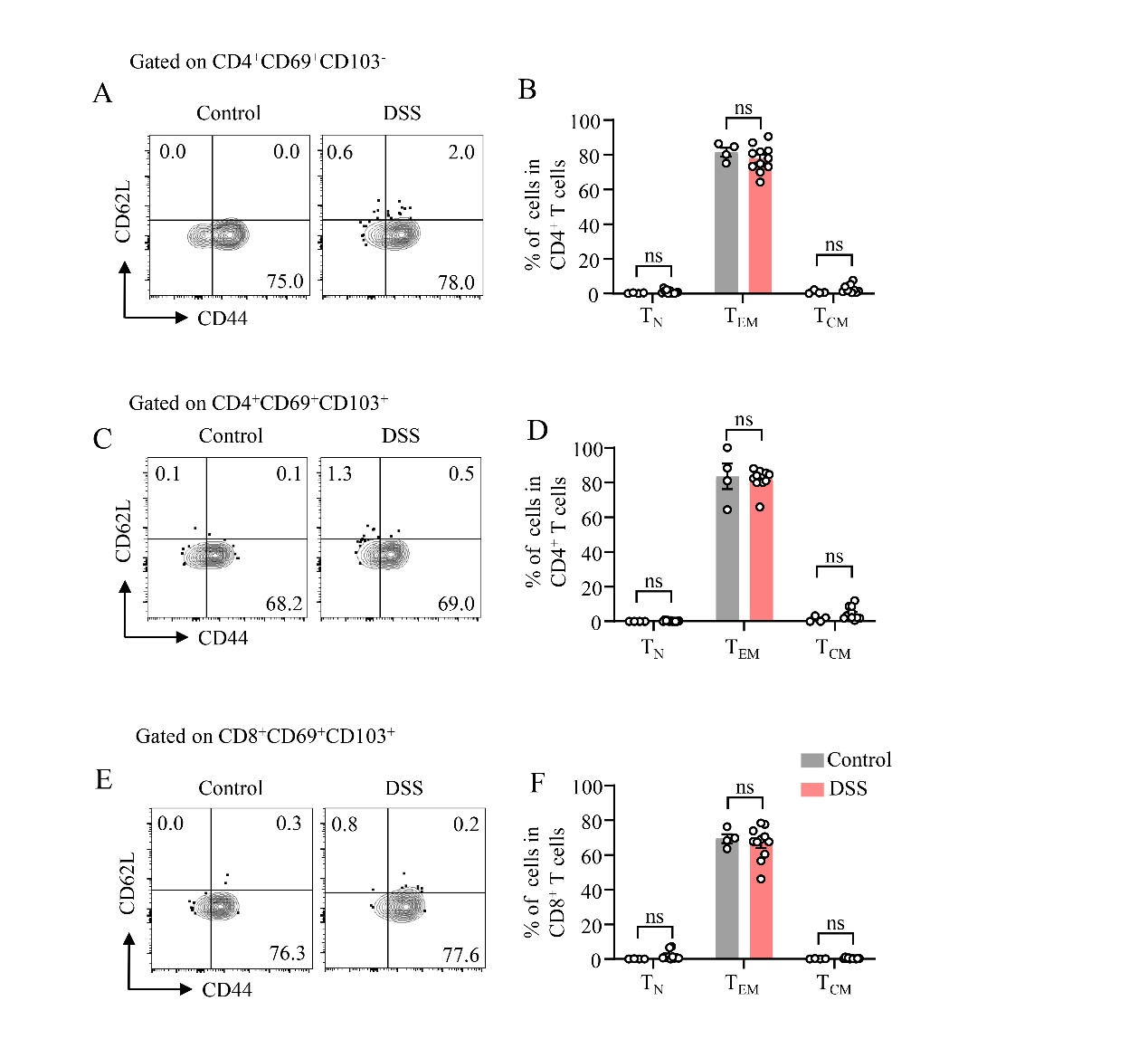


**Supplementary Figure 2. The phenotype of T_RM_ cells in DSS-induced colitis.** DSS-induced colitis was established as in Fig. 1. (A) Representative counter plots for CD44 and CD62L expression in CD4^+^CD69^+^CD103^-^ T cells. (B) Frequencies of CD44^-^CD62L^+^ naïve T cells (T_N_), CD44^+^CD62L^-^ effector memory T cells (T_EM_), and CD44^+^CD62L^+^ central memory T cells (T_CM_) in CD4^+^CD69^+^CD103^-^ T cells measured by flow cytometry. (C) Representative counter plots for CD44 and CD62L expression in CD4^+^CD69^+^CD103^+^ T cells. (D) Frequencies of T_N_, T_EM_, and T_CM_ in CD4^+^CD69^+^CD103^+^ T cells measured by flow cytometry. (E) Representative counter plots for CD44 and CD62L expression in CD8^+^CD69^+^CD103^+^ T cells. (F) Frequencies of T_N_, T_EM_, and T_CM_ in CD8^+^CD69^+^CD103^+^ T cells measured by flow cytometry. ns: not significant.


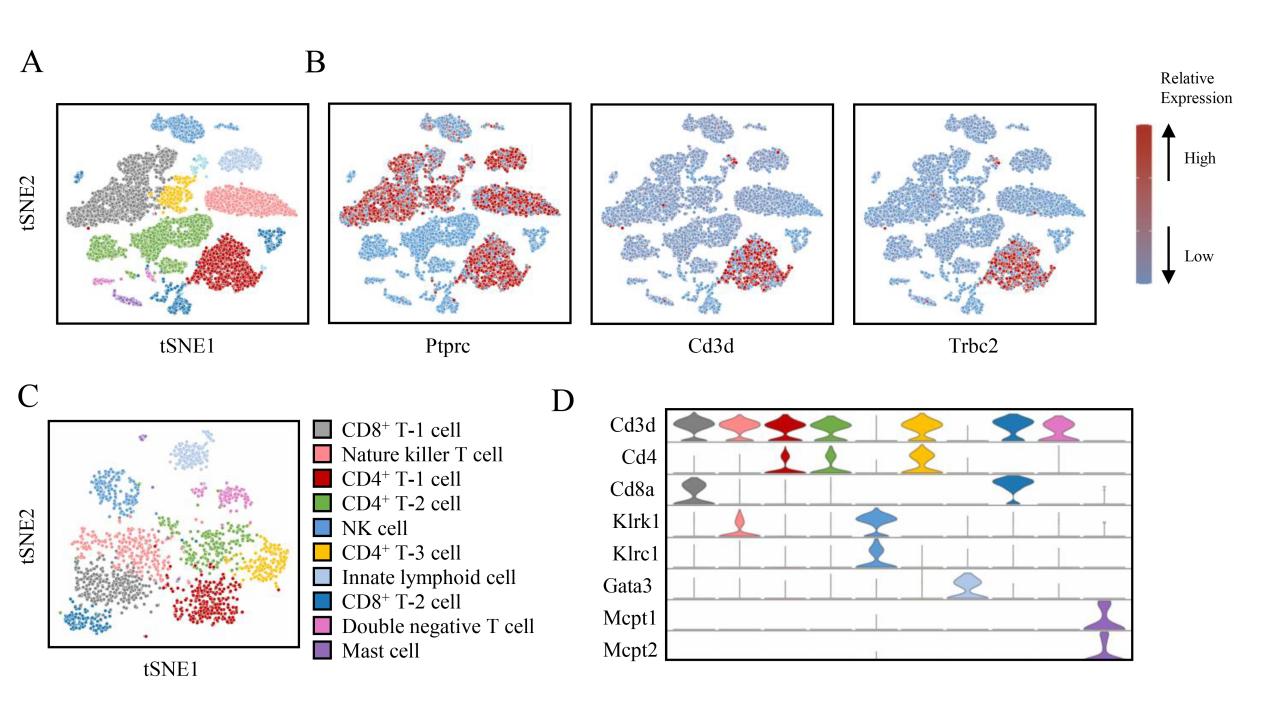


**Supplementary Figure 3. Bio-informative analysis. S**ingle cell RNA-sequencing (scRNA-seq) data of colon cells form DSS-induced colitis were obtained from public data base (Gene Expression Omnibus: GSE148794). The derived matrix was utilized for scRNA-seq analysis properly. (A) t-SNE plot showing 11 clusters of 13566 colonic cells. (B) t-SNE plot showing relative expression of T cell marker genes (*Ptprc, Cd3d, Trbc2*). (C) t-SNE plot showing 10 sub-clusters of the T cell cluster. (D)Violin plots depicting relative expression of highly differential expressed genes of sub-cluster cells, including CD4 T-1, 2, 3 cell (*Cd3d, Cd4*), CD8 T-1, 2 cell (*Cd3d, Cd8*), Nature killer T cell (*Cd3d, Klrk1*), Nature killer cell (*Klrk1, Klrc1*), Group 2 innate lymphoid cell (*Gata3*) and Mast cell (*Mcpt1,Mcpt2*).


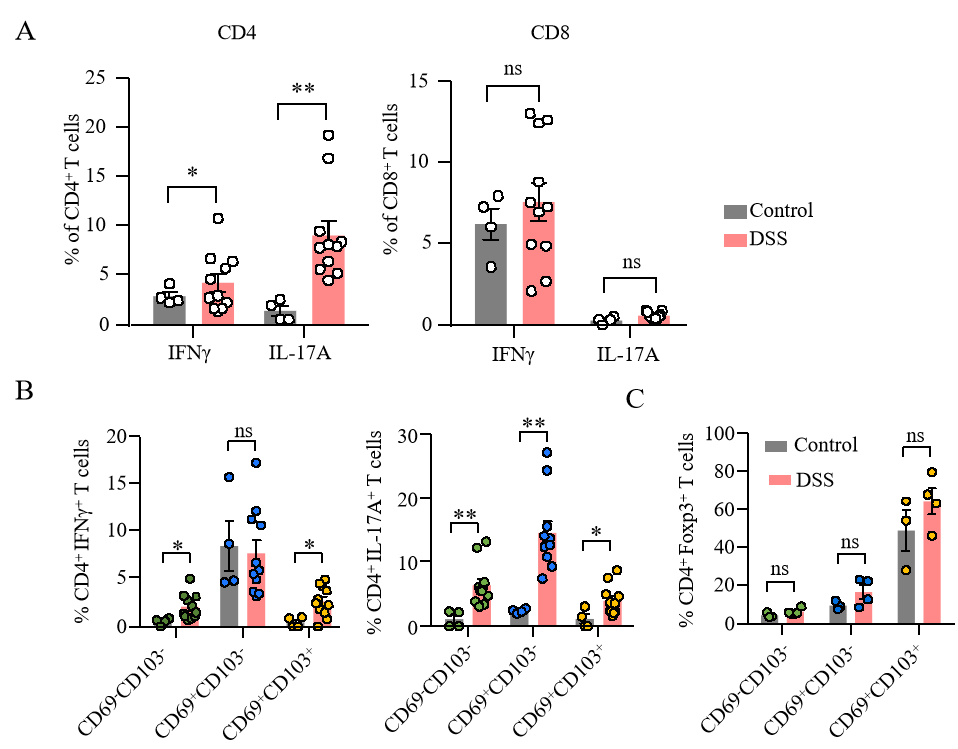


**Supplementary Figure 4. Percentage of CD4^+^ T cell subsets in DSS-induced colitis.** LPMC were isolated from mice with or without DSS-induce colitis. Intracellular cytokine staining of IFNγ and IL-17A was performed in CD4^+^ and CD8^+^ T cells and measured by flow cytometry. (A) Frequencies of IFNγ^+^ and IL-17A^+^ CD4^+^ T cells in total CD4^+^ T cells (left), and frequencies of IFNγ^+^ and IL-17A^+^ CD8^+^ T cells in total CD8^+^ T cells (right) in mice with DSS-induced colitis and control mice. (B) Frequencies of IFNγ^+^ and IL-17A^+^ CD4^+^ T cells in CD4^+^CD69^-^CD103^-^, CD4^+^CD69^+^CD103^-^ and CD4^+^CD69^+^ CD103^+^ subsets in mice with and without colitis. (C) Frequencies of Foxp3^+^ CD4^+^ T cells in CD4^+^CD69^-^CD103^-^, CD4^+^CD69^+^CD103^-^ and CD4^+^CD69^+^CD103^+^ subsets in mice with and without colitis. **p*<0.05, ***p*<0.01 by Student’s T test, ns: not significant.


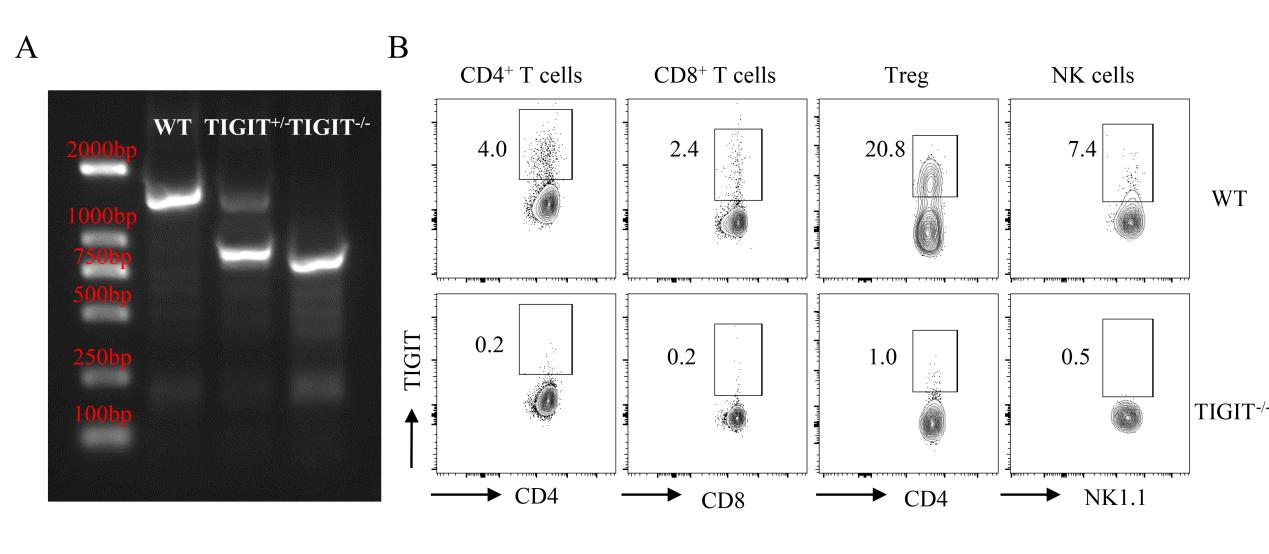


**Supplementary Figure 5. Knockout efficiency of TIGIT^-/-^ mice.** (A) Genomic DNA was extracted from mice and amplified by Polymerase Chain Reaction (PCR). Then agarose gel electrophoresis was performed to separate DNA fragments. Representative TIGIT genotyping results of WT, TIGIT^+/-^, TIGIT^-/-^ mice. WT: 1453bp, TIGIT^+/-^: 1453bp and 730bp, TIGIT^-/-^ 730bp. (B) TIGIT expression in splenocytes from TIGIT^-/-^ or WT mice was measured by flow cytometry. Representative counter plots for TIGIT expression in CD4^+^ T cells, CD8^+^ T cells, Treg cells, NK cells.


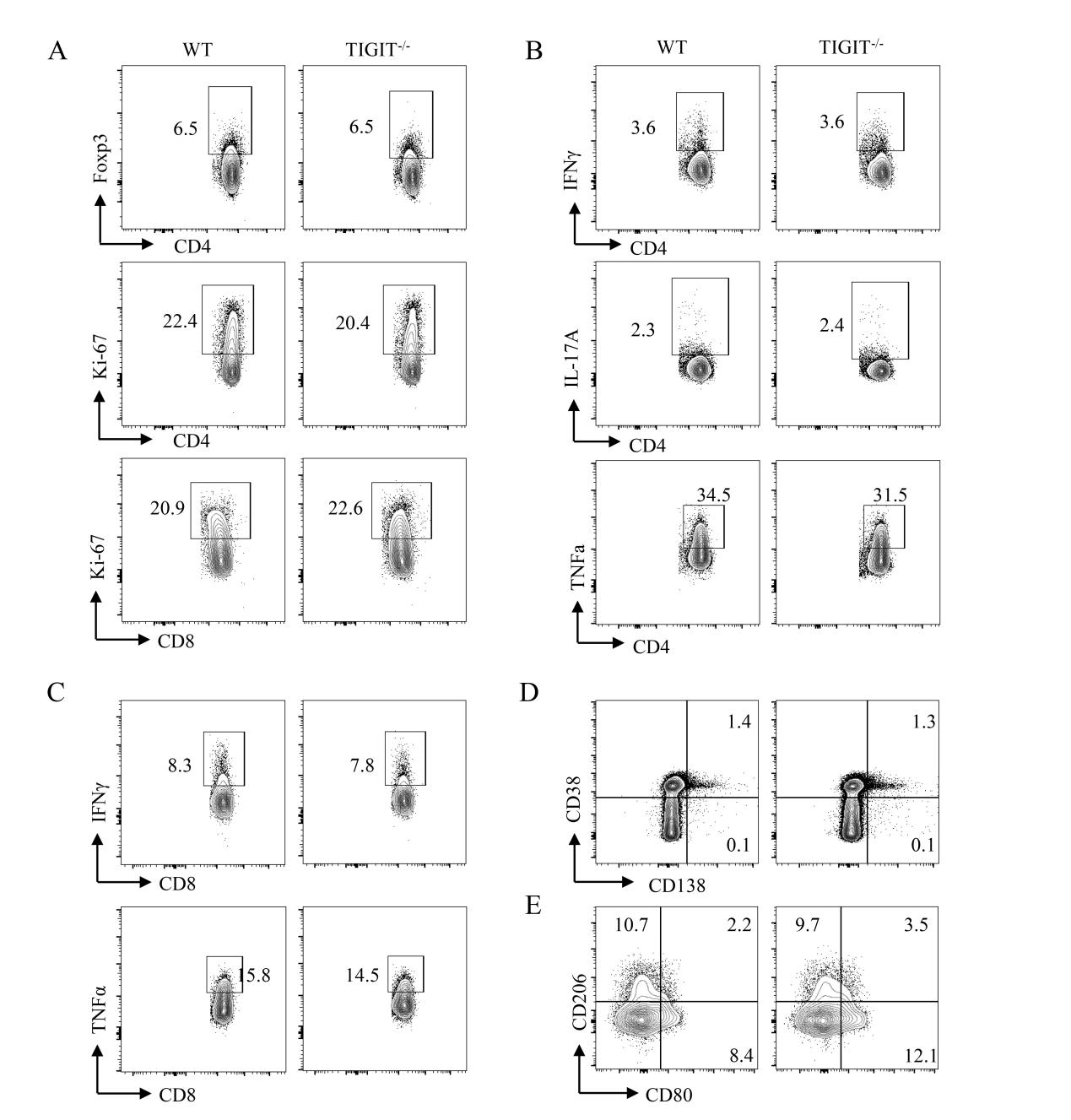


**Supplementary Figure 6. TIGIT deficiency is not sufficient to induced autoimmune response.** (A-F) Single cell suspension was prepared from spleens collected from littermates of TIGIT^-/-^ or WT mice of 8 weeks old. FoxP3, Ki-67 expression (A) and intracellular cytokine expression of IFNγ, IL-17A, TNFα (B-C) in CD4^+^ or CD8^+^ T cells were measured by flow cytometry. Representative counter plots of 3 independent mice. (D) Splenocytes were stained with antibodies against CD38 and CD138 and data was acquired through flow cytometry. Representative counter plots of 3 independent mice. (E) CD80 and CD206 expression in CD11b^+^ monocytes were measured by flow cytometry. Representative counter plots gated on CD11b^+^ cells. Representative counter plots of 3 independent mice.


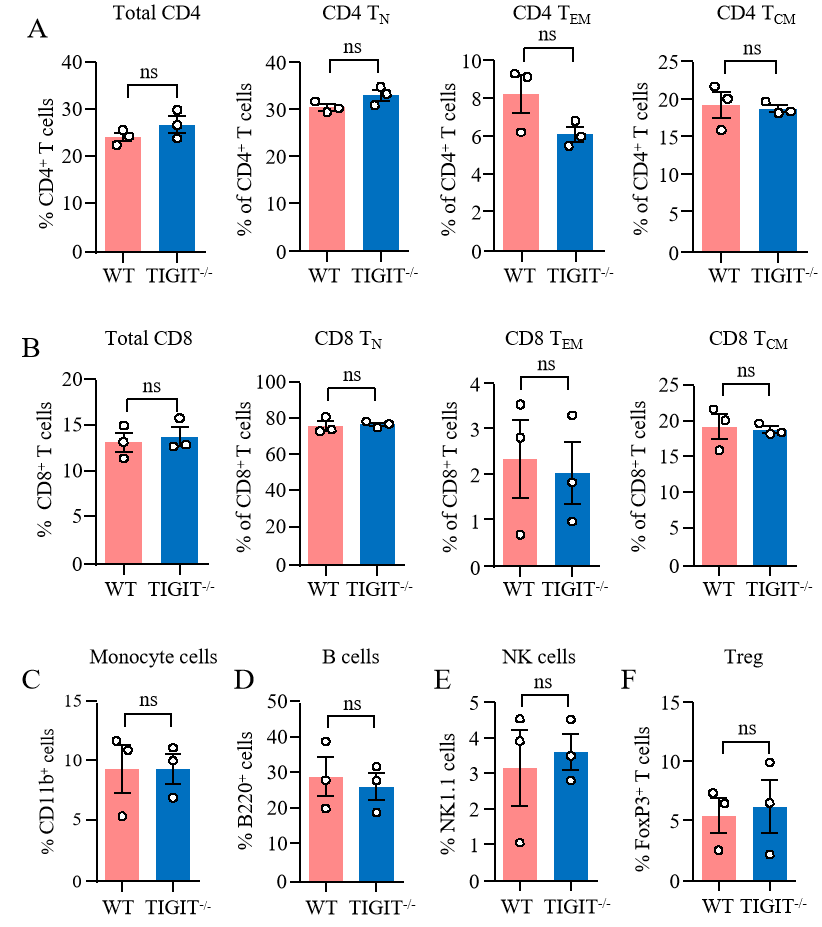


**Supplementary Figure 7. TIGIT deficiency showed no effects on immune homeostasis in the spleen.** Splenocytes from TIGIT^-/-^ or WT mice were stained with antibodies against CD4, CD8, CD44, CD62L, CD11b, B220, NK1.1, Foxp3 and measured by flow cytometry. Gating strategy is shown as in Figure S1. (A) Percentages of total CD4^+^ T cells，naïve CD4^+^ T cells (T_N_), effector memory CD4^+^ T cells (T_EM_) and central memory CD4^+^ T cells (T_CM_). (B) Percentages of total CD8^+^ T cells, CD8 T_N_, CD8 T_EM_ and CD8 T_CM_. (C) Percentages of monocyte, B cells, NK cells and Treg cells. Dara are from 3 independent samples. ns: not significant.


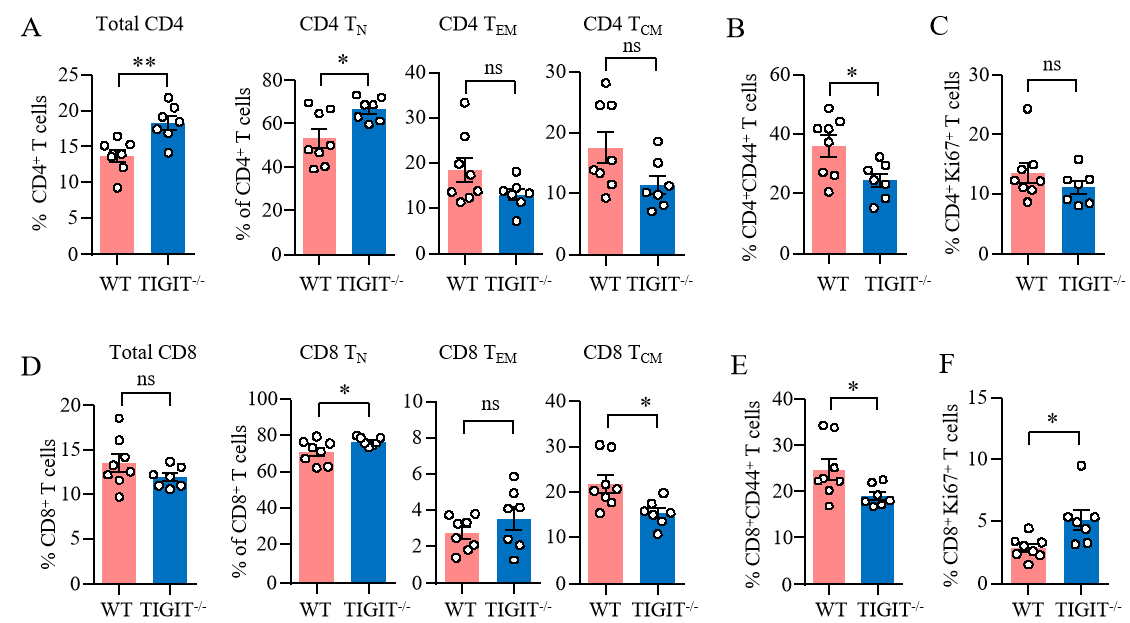


**Supplementary Figure 8. T cell phenotypes in the spleen during DSS-induced colitis.** DSS-induced colitis was established as in Fig. 1 in TIGIT^-/-^ or WT mice. Splenocytes were collected and analyzed by flow cytometry. Gated strategy was performed as in Figure S1. (A) Percentages of total CD4^+^ T cells, naïve CD4^+^ T cells (T_N_), effector memory CD4^+^ T cells (T_EM_) and central memory CD4^+^ T cells (T_CM_) cells in TIGIT^-/-^ or WT mice with DSS-induced colitis. (B) Percentage of CD44^+^ CD4^+^ T cells in TIGIT^-/-^ or WT mice. (C) Percentage of Ki67^+^ CD4^+^ T cells. (D) Percentages of total CD8^+^ T cells, CD8 T_N_, CD8 T_EM_ and CD8 T_CM_. (E) Percentage of CD44^+^ CD8^+^ T cells in TIGIT^-/-^ or wild type mice. (F) Percentage of Ki67^+^ CD8^+^ T cells. WT=8, TIGIT^-/-^=7. **p*<0.05, ***p*<0.01 by Student’s T test, ns: not significant.
